# PDAC-ANN: an artificial neural network to predict pancreatic ductal adenocarcinoma based on gene expression

**DOI:** 10.1186/s12885-020-6533-0

**Published:** 2020-01-31

**Authors:** Palloma Porto Almeida, Cristina Padre Cardoso, Leandro Martins de Freitas

**Affiliations:** 1 Núcleo de Biointegração, Instituto Multidisciplinar em Saúde, Universidade Federal da Bahia, Vitória da Conquista, Brazil; 2 Faculdade Santo Agostinho, Vitória da Conquista, Brazil

**Keywords:** Pancreatic ductal adenocarcinoma, Artificial neural network, Meta-analysis

## Abstract

**Background:**

Although the pancreatic ductal adenocarcinoma (PDAC) presents high mortality and metastatic potential, there is a lack of effective therapies and a low survival rate for this disease. This PDAC scenario urges new strategies for diagnosis, drug targets, and treatment.

**Methods:**

We performed a gene expression microarray meta-analysis of the tumor against normal tissues in order to identify differentially expressed genes (DEG) shared among all datasets, named core-genes (CG). We confirmed the CG protein expression in pancreatic tissue through The Human Protein Atlas. It was selected five genes with the highest area under the curve (AUC) among these proteins with expression confirmed in the tumor group to train an artificial neural network (ANN) to classify samples.

**Results:**

This microarray included 461 tumor and 187 normal samples. We identified a CG composed of 40 genes, 39 upregulated, and one downregulated. The upregulated CG included proteins and extracellular matrix receptors linked to actin cytoskeleton reorganization. With the Human Protein Atlas, we verified that fourteen genes of the CG are translated, with high or medium expression in most of the pancreatic tumor samples. To train our ANN, we selected the best genes (AHNAK2, KRT19, LAMB3, LAMC2, and S100P) to classify the samples based on AUC using mRNA expression. The network classified tumor samples with an f1-score of 0.83 for the normal samples and 0.88 for the PDAC samples, with an average of 0.86. The PDAC-ANN could classify the test samples with a sensitivity of 87.6 and specificity of 83.1.

**Conclusion:**

The gene expression meta-analysis and confirmation of the protein expression allow us to select five genes highly expressed PDAC samples. We could build a python script to classify the samples based on RNA expression. This software can be useful in the PDAC diagnosis.

## Background

The pancreatic ductal adenocarcinoma (PDAC) is the most common pancreatic cancer histological subtype with high mortality due to the lack of symptoms in the initial phase of the disease and its aggressive progression [1, 2]. PDAC development is directly related to *KRAS* overexpression [2, 3], along with the inactivation of the tumor-suppressor genes *CDKN2A/p16* [4], *SMAD4/DPC4* [5] and *TP53* [6, 7]. The *KRAS* activation is considered significant in PDAC progression, and many efforts were made to inhibit its activity [8]; nevertheless, it seems to be undruggable [9]. Data have been presented in the literature over integrated analysis about PDAC genes and proteins, classifying PDAC in different molecular subtypes among patients [10], and through integrated genome analyses that reinforce the participation of *KRAS, TP53, SMAD4,* and *CDKN2A* in a subset of PDAC tumors [11].

Since there is a lack of effective therapies and a low survival rate, the research for new biomarkers and therapies targets in PDAC remains active [12–14]. There are some gene expression changes in pancreatic cancer already described and presented as biological markers. The genes in the ribosome and the spliceosome pathway (ribosomal protein genes Nup170, Nup160, and HNRNPU) were described as potential biomarkers [15]. The meta-analysis of PDAC microarray data could identify five biomarkers (TMPRSS4, AHNAK2, POSTN, ECT2, and SERPINB5) that classified the PDAC and normal samples with sensitivity of 94%, and specificity of 89.6% [16].

Advances in high-performance computing, such as system biology and artificial intelligence (AI) allows integration of data and pattern recognition that generates not only new understating about diseases, but support new targets discovery and biomarkers development for future treatments [17]. The potential to classify the cancer samples using gene expression, methylation information, and AI has been used in other types of cancer studies with promising results. The application of these studies would improve the classification of the samples in tumor diagnosis and subtyping [18–20]. The studies using automatic technics to predict risk/diagnosis had demonstrated a high classification performance, presenting sensitivity > 90% [21–24].

The high number of features coming from microarray gene expression and methylation genomic information used to train AI tumor diagnosis models can give good results in the classification of samples [18, 19], lowering the false-negative rate in training and validation samples. However, the high number of features can make the diagnosis available only for samples with thousands of gene expression values [18]. It has been shown that reducing the number of features can give the same or better results than using thousands of features [25, 26].

The application of AI in pancreatic tumor must improve the early diagnostic and, consequently, the treatment and patient survival. The AI has been used to predict risk/diagnosis using pancreatic image and personal health features [27]. The prediction of pancreatic cancer risk in patients with type 2 diabetes was compared using logistic regression and ANN, again using personal health features and presenting the performance of models predicting the cancer risk factor [24]. There are also AI models to diagnose pancreatic cancer-based in four plasma proteins selected in mass spectra, showing the potential of AI in predicting the status of a sample based on biological markers with high sensitivity (90.9%) and specificity (91.1%) [22]. The Lustgarten Foundation, created to pancreatic cancer research, pointed out the importance of including the AI in the PDAC diagnosis based on MRI and CT scans [28]. The use of new technologies to help pancreatic cancer risk/diagnosis must be pursued, and it would improve patients’ survival. The gene expression changes in pancreatic cancer could be used as biological markers and help in the diagnosis and be used to build a computational model using AI to predict sample status.

In this paper, we performed a meta-analysis of gene expression of public microarray data. We identified a core-gene (CG) group and accessed the protein expression through the Protein Atlas database based on immunohistochemical (IHC) staining images. Clusterization methods were applied to distinguish between normal and PDAC samples. It was selected five genes combining microarray expression and Protein Atlas information. The gene expression information from PDAC and normal samples were used to build an ANN (PDAC-ANN). The PDAC-ANN uses gene expression information to predict the sample status (normal or PDAC) and give the probability of the sample be PDAC. This is the first time gene expression is used to build an ANN model to predict PDAC diagnosis. The results showed here must be verified in a large sample and could be used in the discrimination of samples using these markers. This PDAC-ANN is free software and could be used to improve the diagnosis and help PDAC patients.

## Methods

### Dataset acquisition

The microarray expression data of human healthy and pancreatic cancer tissue were collected from Gene Expression Omnibus (GEO) (https://www.ncbi.nlm.nih.gov/geo) using the search term “pancreatic ductal adenocarcinoma” and selecting mRNA expression profiling by an array. The ten datasets (Table 1) were selected following the criteria: inclusion of (1) studies presenting PDAC/normal samples from the pancreas; exclusion of studies (2) with induced mutations or activated pathways; (3) cells previously exposed to chemotherapy drugs. These criteria ensure that the expression alterations were provided only from the shift normal/disease, and not due to induced mutations in cell lineage or chemotherapy treatment. The datasets were loaded into the R software [39] using the GEOquery package [40]. Ten studies were analyzed to find DEG, and two independent microarray studies provided samples to validate the CG derived from the meta-analysis.

**Table 1:** Characteristics of studies used in the meta-analysis

Accession number	Study	Array platform	Differentially expressed genes		Samples	
			Upregulated	Downregulated	Tumor	Normal
GSE23397	*	Affymetrix Human Exon 1.0 ST Array	4031	870	15	6
GSE28735	[29]		245	146	45	45
GSE41368	[30]		1200	462	6	6
GSE32676	[31]	Affymetrix Human Genome U133 Plus 2.0 Array	686	319	25	7
GSE71989	[32]		3052	661	13	8
GSE15471	[33]		1546	227	39	39
GSE62165	[13]	Affymetrix Human Genome U219 Array	2638	1266	118	13
GSE43795	[34]	Illumina HumanHT-12 V4.0 expression beadchip	1978	1343	6	5
GSE71729	[35]	Agilent-014850 Whole Human Genome Microarray 4x44K G4112F	285	175	145	46
GSE60979	[36]	Agilent-028004 SurePrint G3 Human GE 8x60K Microarray	1365	1336	49	12
Total					461	187
GSE16515	[37]	Affymetrix Human Genome U133 Plus 2.0 Array	–	–	36	16
GSE62452	[38]	Affymetrix Human Gene 1.0 ST Array			69	61
Total					105	77

* No publication available

- Analysis of differentially expressed genes was not applied to the validation dataset

### Data processing

Non-specific filtering and identification of differentially expressed genes (DEG) were applied to each dataset coming from the same GEO series using packages from Bioconductor [41]. Briefly, the package genefilter was used to remove the genes with no expression variation among samples [42], followed by the collapse of multiple probe measurements of a given gene into a single gene measurement in package WGCNA [43]. The limma package [44] was used to identify the DEG through a t-test. We considered DEGs when log2 fold change (log2FC) was ≥1 and adjusted *p*-value by false discovery rate (FDR) ≤ 0.05 [45, 46].

### Core-gene analysis

The DEG frequency among the microarray studies was retrieved, and those shared by all microarray studies were considered as the CG. The CG expression values were standardized, applying the method ${X}^{\prime }=\frac{X-\overline{X}}{sd}$, where *X* represents the expression values, $\overline{X}$ the gene expression average, and *sd* standard deviation [47]. This standardization was followed by a min-max data rescale, for each gene transforming all values to [0, 1] range. Thus, restricting values from different studies to the same range [48]. The CG standardized values were used in the Principal Component Analysis (PCA) and the hierarchical clustering in order to check the clustering of the samples from all datasets based on the CG expression values.

### Data validation

The IHC staining images and the protein expression data from pancreatic cancer tissue were used as validation of the CG. Protein expression data were obtained from the Human Protein Atlas (HPA) (www.proteinatlas.org) [49]. The number of IHC staining images present in HPA categories (high, medium, low, not detected) was counted to each gene. These IHC staining images were used as validation of protein expression when the number of high plus medium stating images was ≥75%.

We also investigated a validation using the CG mRNA standardized values in two independent datasets (GSE16515 and GSE62452). We applied the hierarchical clustering/heatmap, PCA, and artificial neural network to the validation samples to evaluate its capability to differentiate tumor and normal groups using the microarray information.

### Neural network sample classification

**Fig. 1:**
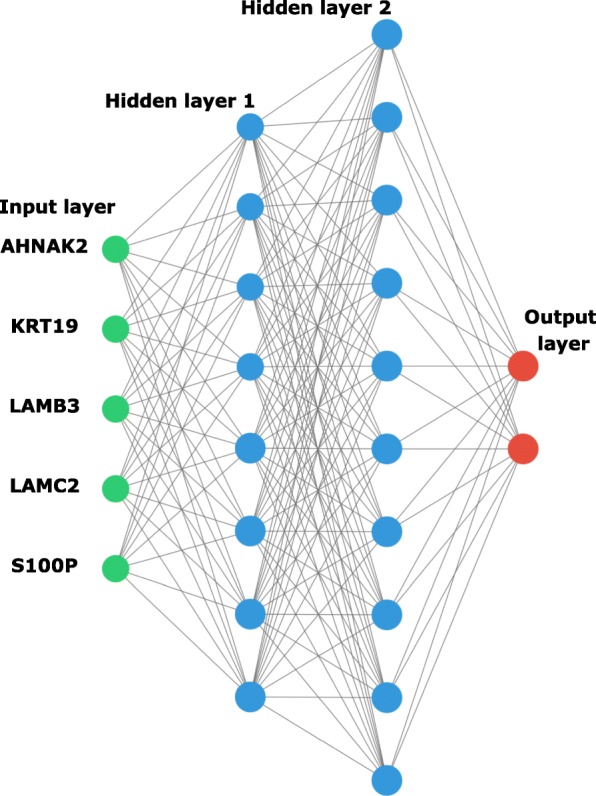
Artificial neural network architecture. A graphical representation of a fully connected artificial intelligence algorithm (PDAC-ANN). PDAC-ANN is a set of mathematical equations; in each layer, it transforms expression values up to the last layer. The expression values from AHNAK2, KRT19, LAMB2, LAMC2, and S100P genes are data inserted in the input layer (green neurons), the hidden layers (blue neurons) process the expression values, and the output layer (red neurons) give the classification in normal or PDAC sample as a probability

We build an artificial neural network (ANN) using python to classify the sample in normal or tumor samples. The ANN was trained using normalized gene expression values [0, 1] from the five genes with the highest AUC among the CG confirmed by HPA (Fig. 1). We explore the performances of 90 network architectures with one input layer with five nodes (input neurons to gene expression values), one or two hidden layers varying the number of nodes from 2 to 10, and two output nodes, giving the normal and PDAC probability. Each network architecture was trained 30 times, and we took the mean accuracy in the train set to evaluate the classification performance. We used a learning rate of 0.05, 100 epochs during training, relu and softmax as activation functions for internal and output node, respectively. The network weights were randomly initialized with values between [− 1, 1], and bias with value 1.

### Statistics and analysis

Results are presented as a bar plot, representing the protein expression as indicated in the HPA, and PCA or heatmap, representing the variation and clusterization among the samples based on mRNA gene expression. The IHC results graph, PCA, and heatmap were produced using the R statistical computational language [50] and the ggplot2 package [51]. The statistical tests (ROC, AUC, and DEG) and *p*-value correction were performed using the R language. The sensitivity, sensibility, and accuracy were calculated using python language, getting the results from the confusion matrix in training and validation datasets.

## Results

### Differentially expressed genes in meta-analysis

To profile differentially expressed genes in PDAC, we performed a meta-analysis of microarray data available in Table 1. We collected and compared 463 tumor samples to 187 normal tissues. We have identified 10,861 unique DEG, where 7028 were upregulated and 3833 downregulated genes (log2FC = 1; adj. *p*-value ≤0.05) (Additional file 1: Table S1). The ten studies shared 40 DEG (CG), where 39 were upregulated, and one downregulated (Table 2).

**Table 2:** Description of the core-genes involved in the PDAC biological process

Gene symbol	Gene name	Gene symbol	Gene name
*Upregulated*			
AHNAK2	AHNAK nucleoprotein 2	KRT19	keratin 19
ANLN	anillin actin binding protein	LAMA3	laminin subunit alpha 3
ANO1	anoctamin 1	LAMB3	laminin subunit beta 3
ASPM	abnormal spindle microtubule assembly	LAMC2	laminin subunit gamma 2
CAPG	capping actin protein, gelsolin like	LCN2	lipocalin 2
CEACAM5	carcinoembryonic antigen related cell adhesion molecule 5	MET	MET proto-oncogene, receptor tyrosine kinase
CEACAM6	carcinoembryonic antigen related cell adhesion molecule 6	NQO1	NAD(P)H quinone dehydrogenase 1
COL10A1	collagen type X alpha 1 chain	OAS1	2′-5′-oligoadenylate synthetase 1
CXCL5	C-X-C motif chemokine ligand 5	S100A14	S100 calcium binding protein A14
DKK1	dickkopf WNT signaling pathway inhibitor 1	S100P	S100 calcium binding protein P
FXYD3	FXYD domain containing ion transport regulator 3	SERPINB5	serpin family B member 5
GABRP	gamma-aminobutyric acid type A receptor pi subunit	SLC2A1	solute carrier family 2 member 1
GCNT3	glucosaminyl (N-acetyl) transferase 3, mucin type	SLC44A4	solute carrier family 44 member 4
GJB2	gap junction protein beta 2	SLC6A14	solute carrier family 6 member 14
GPRC5A	G protein-coupled receptor class C group 5 member A	SLPI	secretory leukocyte peptidase inhibitor
GPX2	glutathione peroxidase 2	TCN1	transcobalamin 1
IFI27	interferon alpha inducible protein 27	TFF1	trefoil factor 1
ITGA2	integrin subunit alpha 2	TMC5	transmembrane channel like 5
ITGA3	integrin subunit alpha 3	TMPRSS4	transmembrane protease, serine 4
		TSPAN1	tetraspanin 1
*Downregulated*			
AOX1	aldehyde oxidase 1		

The CG showed a profile of upregulated genes functions related to cell membrane-ECM interaction (*LAMA3*, *LAMB3, LAMC2*), cytoskeleton interaction/calcium management *(GCNT3, ANLN, S100A14, S100P*), and structural integrity of epithelial cells (*ITGA2, ITGA3, KRT19*). Most of the genes reinter the importance of the ECM interaction and cellular morphology in carcinogenic processes in PDAC. The AOX1 was the only downregulated gene in PDAC compared to normal samples. The AOX1 was already detected as downregulated in other PDAC studies [52, 53], and this corroborates the result presented here.

### Immunohistochemical staining images validation

**Fig. 2:**
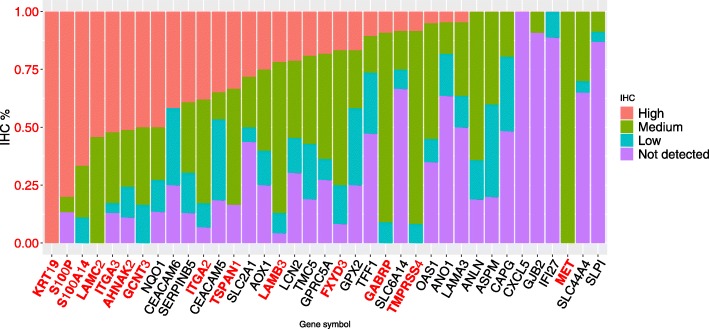
Variation in protein expression data from the GC list retrieved from immunohistochemical staining images in HPA. The protein expression data shows that 14 genes have more than 75% of images with high plus medium expression in pancreatic cancer, evidencing the expression of predicted core-genes in the pancreatic tissue. The genes with protein expression confirmed in IHC staining images were highlighted in red. Data credit: Human Protein Atlas

**Fig. 3:**
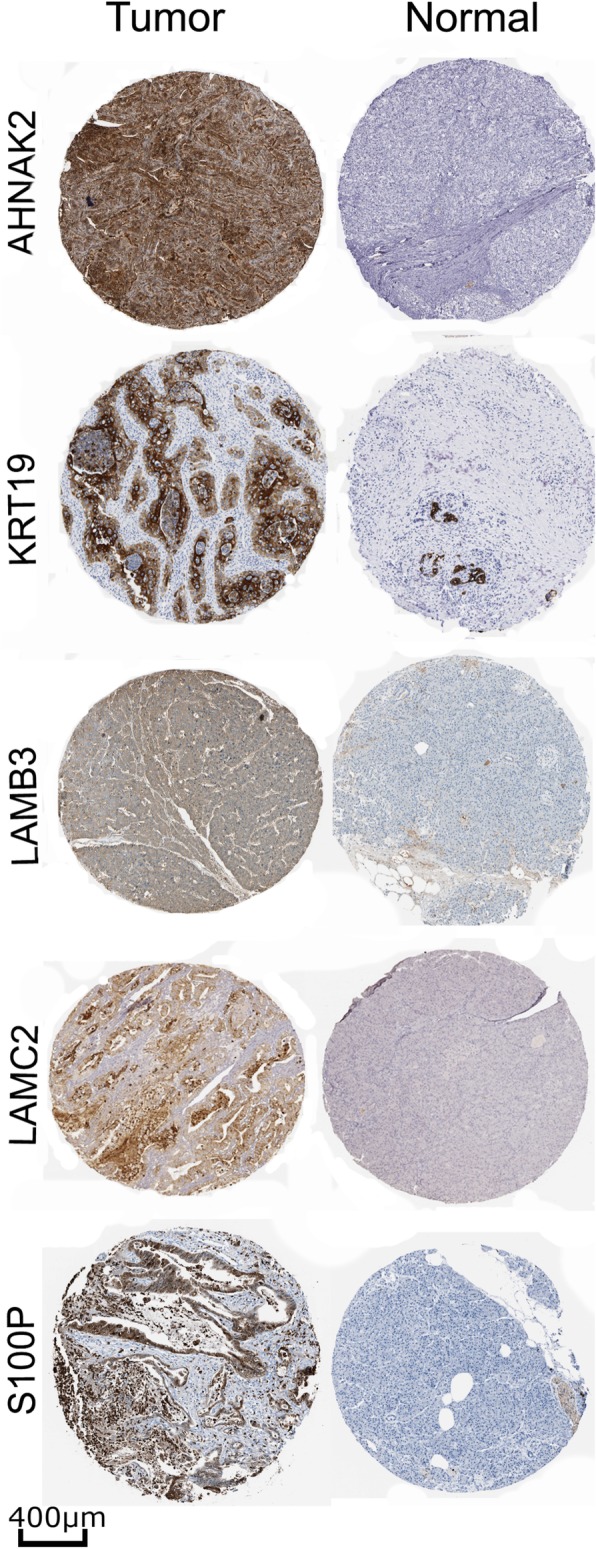
Representative immunohistochemistry staining of AHNAK2, KRT19, LAMB2, LAMC2, and S100P in Pancreatic Ductal Adenocarcinoma (Tumor) and normal pancreatic tissue (Normal). The proteins presented more than 75% of images with high plus medium expression in HPA. Scales bars represent 400 μm. Image courtesy of Human Protein Atlas

To determine whether the CG is also present as proteins expressed in PDAC, we investigated the expression of these genes in HPA. This analysis could confirm the protein expression from many of the CG list using information from IHC staining images. The protein expression data from the CG showed that 14 genes have more than 75% of images with high or medium expression in pancreatic cancer (Fig. 2). More than 75% of IHC images stained for KRT19 and S100P showed high expression values of these genes at the protein level (Fig. 3), from a set of 23 and 12 images in HPA, respectively.

The genes CXCL5, GJB2, IFI27, and SLPI, have a low or no expression detected in at least 90% of samples, not corroborating with the CG list. The protein AOX1 presents a different expression between the RNA and protein levels. The AOX1 protein is highly expressed in some samples (60%) and low or not detected in others (40%) in HPA. There were three proteins (COL10A1, DKK1, and TCN1) with no information in HPA; thus, it is not possible to report about the protein expression in pancreatic cancer. All these data show essential genes in PDAC highly expressed in proteins level, confirming 14 genes from the CG in pancreatic cancer.

### Classification of the merged samples in tumor and control using PCA and hierarchical clustering

We performed hierarchical clustering of the samples/genes and a PCA analysis of the samples to evaluate how different the gene expression is among the samples and how the samples cluster. The PCA showed variation in the expression in a continuous manner, and some PDAC samples mixed with normal samples. Although this continuum between the normal and PDAC samples, the PCA plot has a region with only PDAC samples, indicating more specific gene expression in PDAC. The PCA result indicates a difference in the CG expression enough to classify the samples in normal and PDAC; however, the PCA does not predict the label of the sample (Additional file 2: Figure S1). The continuum and mixture of samples indicate that some samples present a different gene expression pattern and are closer to samples from the other group.

**Fig. 4:**
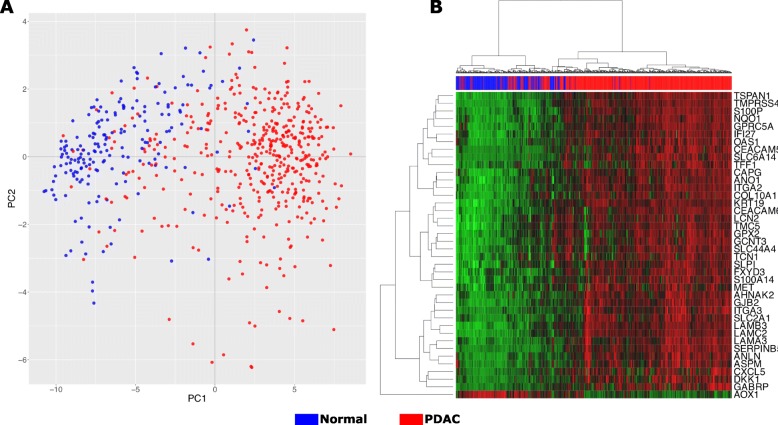
PCA and hierarchical analysis of the merged data set into one data. **a**. PCA analysis clearly showed two distinct groups corresponding to normal and tumor samples. **b**. Clustering analysis. The red band indicates the PDAC samples with similar gene expression on 40-core-gene, and the blue band indicates the normal samples

The hierarchical clustering, performed using CG expression standardized values from all ten datasets, reveals the presence of two groups, and it is possible to check the error of the sample classification (Fig. 4). The standardized CG expression values were able to classify the data into two groups in a continuous manner, once more indicating that these groups exhibit distinctly cellular processes and functions. The hierarchical clustering showed the ratio Normal Classified/Normal = 85.5 and Tumor Classified/Tumor = 85.6.

The methodology was also applied to independent datasets (GSE16515 and GSE62452) to validate the CG found in the meta-analysis. The CG expression values from these independent datasets produced similar results in both PCA and heatmap hierarchical clustering analysis (Additional file 2: Figure S1). The PCA and heatmap showed that CG could classify the data in two groups of normal and tumoral samples, which suggest that the CG maps central process in PDAC. Together, these results indicate that the CG expression can distinguish the groups normal from PDAC samples, with different functional/cellular processes expressed by this condition, and this points to CG list as critical genes in PDAC that could be used to classify the samples and improve diagnosis.

### Neural network sample classification

The best neural network architecture had a mean accuracy of 88.1 and 85.71% in the train and test set respectively; the architecture has five input neurons, eight and ten neurons in the next two hidden layers, and two output. We selected the best-trained network with this architecture with an accuracy of 89.66. We examined the classification performance in the validation dataset using the f1-score, which summarize the precision and recall measurements (Table 3). The f1-score was 0.83 for the normal samples and 0.88 for the PDAC samples, with an average of 0.86. The confusion matrix showed that the number of true negatives (normal) was 64/77, while the number of true positives is 92/105 (Table 4).

**Table 3:** Classification report of the validation test set

	Precision	Recall	F1-score	Support
Normal	0.83	0.83	0.83	77
Tumor	0.88	0.88	0.88	105
Avg/total	0.86	0.86	0.86	182

**Table 4:** Confusion matrix of the training and validation test samples

		Actual normal	Actual tumor
Training	Classified normal	169	49
	Classified tumor	18	412
		Specificity = 90.4	Sensitivity = 89.4
Test	Classified normal	64	13
	Classified tumor	13	92
		Specificity = 83.1	Sensitivity = 87.6

## Discussion

We performed a meta-analysis of mRNA expression data recovered from public datasets, intending to investigate the profile of molecular alterations in pancreatic ductal adenocarcinoma and use this information to build an ANN predictor. Comparing 461 tumor samples to 187 normal tissues, we were able to observe a central group of genes linked to carcinogenic processes, labeled core-genes. Further, we investigated the protein expression with immunohistochemistry information recovery from HPA and validated with two independent microarrays through hierarchical clustering and PCA. The late diagnosis and high mortality rate in PDAC patients demand better tools to improve the diagnosis. Currently, the gold standard blood-based biomarker for PDAC diagnosis is the CA 19–9 [54]. However, CA 19–9 lacks the sensitivity for the early detection and also has a poor predictive value in asymptomatic patients [55–57]. Imaging screening, like magnetic resonance imaging (MRI) and computed tomography (CT), while accurate, is expensive and uncomfortable [58]. Thus, the precise selection of biomarkers can increase the accuracy in the diagnosis of PDAC as well as provide a cheaper diagnostic method with a lower invasion.

We performed a validation of the CG through the IHC images retrieved from HPA, and our results indicated a list of possible PDAC biomarkers. Furthermore, we presented a biomarker often used for PDAC diagnosis, the carcinoembryonic antigen-related cell adhesion molecule 5 (CEACAM5, also known as CEA). The CEACAM5 has been pointed as the second serum biomarker most used clinically for detecting PDAC [28].

We confirmed the expression of 14 genes from CG with high expression in the protein level. These proteins are involved in many functions in cancer biology. For instance, the most expressed protein, keratin 19 (KRT19), is a structural protein of epithelial cells, with expression in a subset of pancreatic cells [59]. The KRT19 was already described as a possible biomarker for PDAC, and patients with upregulation of KRT19 presents poor differentiation, large tumor size, lymph node metastasis, and invasion [60]. In other gastrointestinal cancers, clinical-pathological analyses revel KRT19 correlated with metastasis, tumor size, microvascular invasion, decreased tumor differentiation, and also conferred an invasive phenotype [60].

The laminin subunit gamma 2 (LAMC2) and beta 3 (LAMB3) proteins were shown to be upregulated in PDAC samples using microarray, immunohistochemical analyses, and biomarkers for diagnosis and prognosis integrating a multigene panel [61–63]. Proteomic analysis pointed the LAMC2 as a potential biomarker for PDAC, being upregulated with an mRNA fold of 8.36. The serum concentration of LAMC2 in patients with PDAC was ∼ 3.5-fold higher from benign and normal samples, indicating this gene as a promising biomarker [64]. PDAC patients expressing the high amount of LAMC2 have a poor prognosis [63], reinforcing this gene as a putative biomarker for diagnosis or prognosis. The LAMB3 is involved in the first stage and progression of PDAC, promotion of cell proliferation, inhibition of apoptosis, and is also involved in metastatic PDAC [63, 65]. These results showed the critical association of LAMC2 and LAMB3 with PDAC and highlighted them to be used as therapeutic targets in PDAC treatment [62, 65].

The AHNAK Nucleoprotein 2 was already reported as a PDAC biomarker with tissue-based evidence, thus, confirming AHNAK2 expression in protein level [16, 61, 66]. In our analysis, AHNAK2 was highly expressed in 23 of 45 PDAC samples, as indicated in the HPA results. The AHNAK2 function in PDAC in poorly described; however, another similar *AHNAK* gene is involved with migration and the epithelial-mesenchymal transition, indicating the AHNAK2 may be involved in these processes as well [67]. AHNAK2 high expression is associated with PDAC poor prognosis and is also expressed in bladder and kidney cancer [68, 69].

The S100 Calcium Binding Protein P was reported as a useful biomarker for PDAC based on IHC with expression already reported in gastric and bladder cancer [70]. In PDAC, S100P is expressed in precursor lesions and is involved with tumor growth and invasion [71, 72]. We showed that S100P was one of the three proteins detected with high expression based on IHC in HPA (6 of 9 samples). S100P was studied to discriminated normal and PDAC samples using a higher concentration in duodenal fluid in patients with PDAC compared with the control group, presenting an AUC of 0.71 for detecting PDAC [73]. Our results showed an AUC of 0.92 for S100P using mRNA expression (Additional file 4: Figure S2). A meta-analysis study showed S100P as a potential biomarker to discriminate PDAC samples using RT-PCR or IHC and reported a sensitivity and a specificity of 0.87 and 0.88, respectively [74].

In addition to IHC validation, the CG expression values were tested in independent samples. The PCA and the heatmap hierarchical clustering analysis indicated that CG plays a central process in PDAC and is capable of classifying the data in two groups of normal and tumoral samples. Although there were core regions with a higher number of normal or PDAC samples, some PDAC samples presented gene expression similar to normal samples and were misplaced in PCA. The microarray analysis using PCA already showed that higher dimensionality of the PCA, beyond the first two or three dimensions, can hold valuable information, thus limiting the PCA interpretations [75, 76]. The CG in these set of samples present a different pattern, and it is not possible correctly assigning them based on this gene expression. The use of ANN could increase the correct classification, leading to higher sensitivity. Even though, in the validation dataset, 13 samples in each group were incorrectly classified, pointing a limitation.

We used five genes to develop an ANN sample classifier. We achieve sensitivity and specificity of 87.6 and 81.8%, respectively, applying our ANN classifier in the test set. The development of automatic classifiers based on artificial intelligence can aid the PDAC diagnosis. Five possible PDAC biomarkers were already pointed (FAIM3, IRANK3, DENND2D, PLBD1, AGPAT) based on gene expression, achieving a combined sensitivity of 100% and specificity of 94% [77]; however, no automatic classification was produced. These five genes were pointed as potential biomarkers in PDAC diagnosis. Here, we not only pointed five genes independently differentially expressed among datasets but also created an automatic tool to classify the samples and give the probability of being normal or PDAC. In contrast with the list of five differentially genes reported by Irigoyen et al. 2018 [77], the CG list reported here did not include any of these genes.

In another study, artificial intelligence was developed with support vector machines (SVM) to classify samples using PDAC gene expression information of five genes (TMPRSS4, AHNAK2, POSTN, ECT2, and SERPINB5). Using different genes, our ANN has different results compared with the PDAC SVM classifier that showed validation dataset sensitivity 88.89–97.22% and specificity of 85.7–96.5% [16]. The variation of sensitivity and specificity indicates that the SVM classifier has better performance in some datasets. While our ANN was applied to all validation samples at once and the values of sensitivity and specificity are closer to the potential of classifying PDAC samples based on gene expression. The datasets used in both works are different, with this in mind, sample preparation or microarray technologies (Affymetrix and Illumina) could be possible explanations to different gene lists. Furthermore, the use of ten datasets here in contrast with two datasets by Irigoyen et al. 2018 [77] could also produce different results. Another explanation for these differences in the gene list presented here could be due to PDAC subtypes already studied in gene expression and clinical level [10].

## Conclusions

The results indicated that PDAC presents a 40-core gene signature, with 39 genes upregulated and one downregulated. Among these upregulated genes, many are related to cell adhesion, migration, and extracellular matrix-receptor interaction; the downregulated gene is associated with pancreatic functions. Immunohistochemical analyses confirm the overexpression at the protein level of 14 genes, validating our analysis. The five most overexpressed genes were related to tumor differentiation, cell migration, and metastasis. The PDAC-ANN trained using gene expression information could classify the samples in normal and PDAC with an f1-score of 0.82 and sensitivity 87.6. The ANN diagnosis tool can only be used when the gene expression information from AHNAK2, LAMB3, LAMC2, KRT19, and S100P are available, in addition to min-max gene expression values rescaling. The PDAC-ANN is a free tool that can support in the pancreatic ductal adenocarcinoma diagnosis.

## Availability and requirements

Project name: Pancreatic ductal adenocarcinoma artificial neural network (PDAC-ANN).

Project home page: https://github.com/freitasleandro/PDAC-ANN

Operating system(s): e.g. Platform independent.

Programming language: Python 3.7.

Other requirements: pandas, numpy, sklearn, keras, tensorflow, argparse.

License: GNU GPL v3.0.

Any restrictions to use by non-academics: licence needed.

## Supplementary information

**Additional file 1: Table S1.** Microarray gene expression results from the six studies here. Excel file with information of spot ID, adjusted *p*-value ≤0.05, |log2FC| ≥ 1,Gene symbol, Gene.ID (ENTREZ_GENE_ID).

**Additional file 2: Figure S1.** PCA and hierarchical analysis of the CG expression values from GSE16515. (a) The CG could produce similar results in both PCA and (b) heatmap hierarchical clustering analysis. The CG can classify the data into two groups of normal and tumoral samples.

**Additional file 3: Table S2.** Excel file with links to protein expression level from the CG and links to immunohistochemical images available in HPA.

**Additional file 4: Figure S2.** Receiver operating characteristic (ROC) curve for the five genes selected to train the ANN. The AUC was used to select the genes to train the ANN. The genes selected were AHNAK2 (92.2), KRT19 (92.6), LAMB2 (93.3), LAMC2 (91.8), and S100P (92.3). The AUC for each gene is presented in the parentheses.

## Data Availability

The datasets analyzed during the current study are available in the GEO repository under accession numbers GSE23397, GSE28735, GSE32676, GSE41368, GSE43795, GSE71989, GSE15471, GSE62165, GSE71729, GSE60979, GSE62452, and GSE16515. The immunohistochemistry images and antibody staining levels are available in HPA (https://www.proteinatlas.org). Links to antibody staining levels and images are submitted as Additional file 3: Table S2.

## Abbreviations

AI: Artificial intelligence
ANN: Artificial neural network
AUC: Area under the curve
CAPES: Coordination of Improvement of Higher Education Personnel
CG: Core-genes
CT: Computed tomography
DEG: Differentially expressed genes
ECM: Extracellular matrix
GEO: Gene Expression Omnibus
IHC: Immunohistochemical
MRI: Magnetic resonance imaging
mRNA: messenger RNA
PCA: Principal component analysis
PDAC: Pancreatic ductal adenocarcinoma
PNPD: National Postdoctoral Program
ROC: Receiver operating characteristic
SVM: Support vector machine
HPA: Human Protein Atlas
