# Supplementary figures and images for: PDAC-ANN: an artificial neural network to predict pancreatic ductal adenocarcinoma based on gene expression
Source: BMC Cancer. 2020 Jan 31;20:82. doi: 10.1186/s12885-020-6533-0 (PMC6995241; doi:10.1186/s12885-020-6533-0)

**A**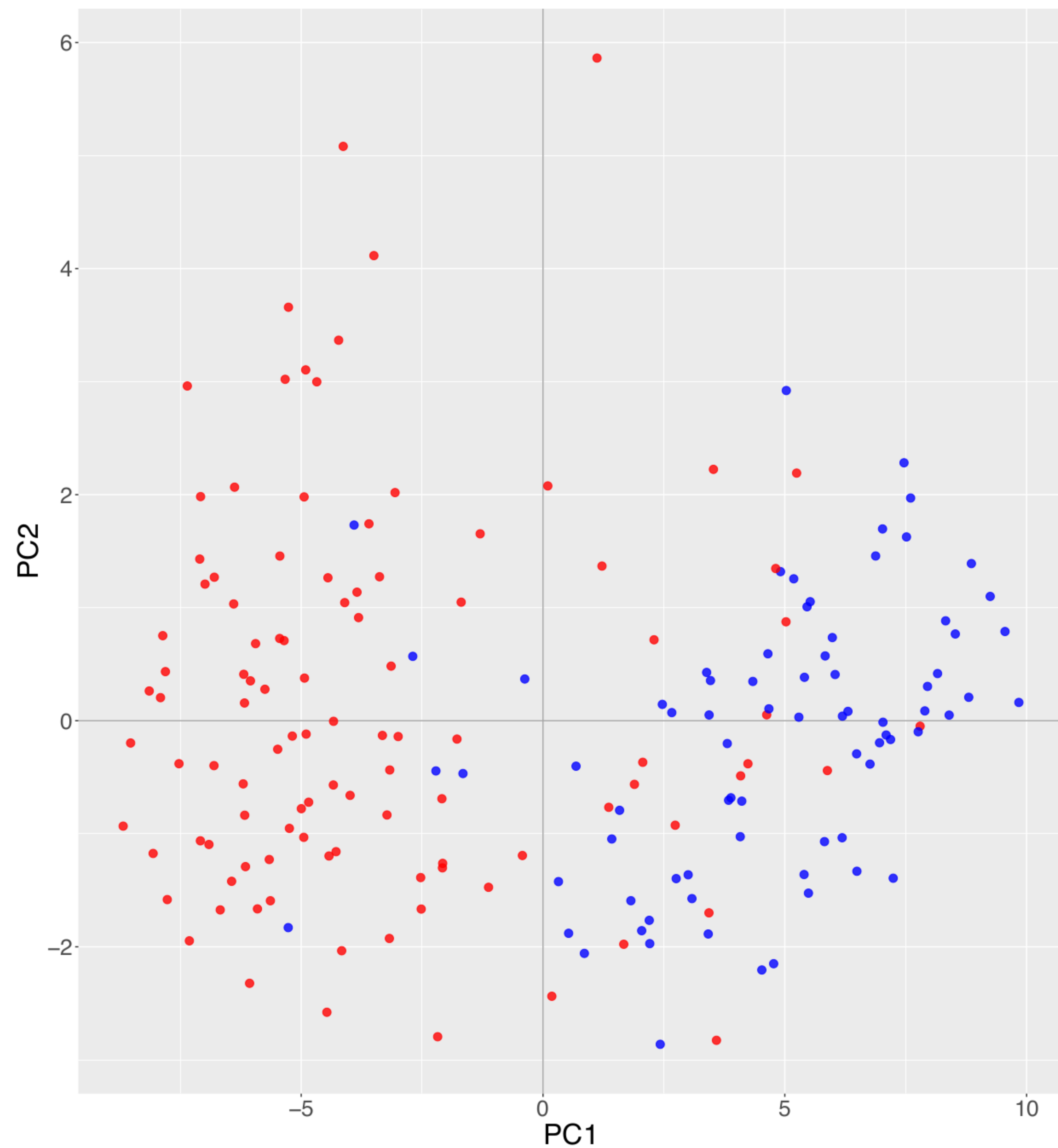**Normal****B**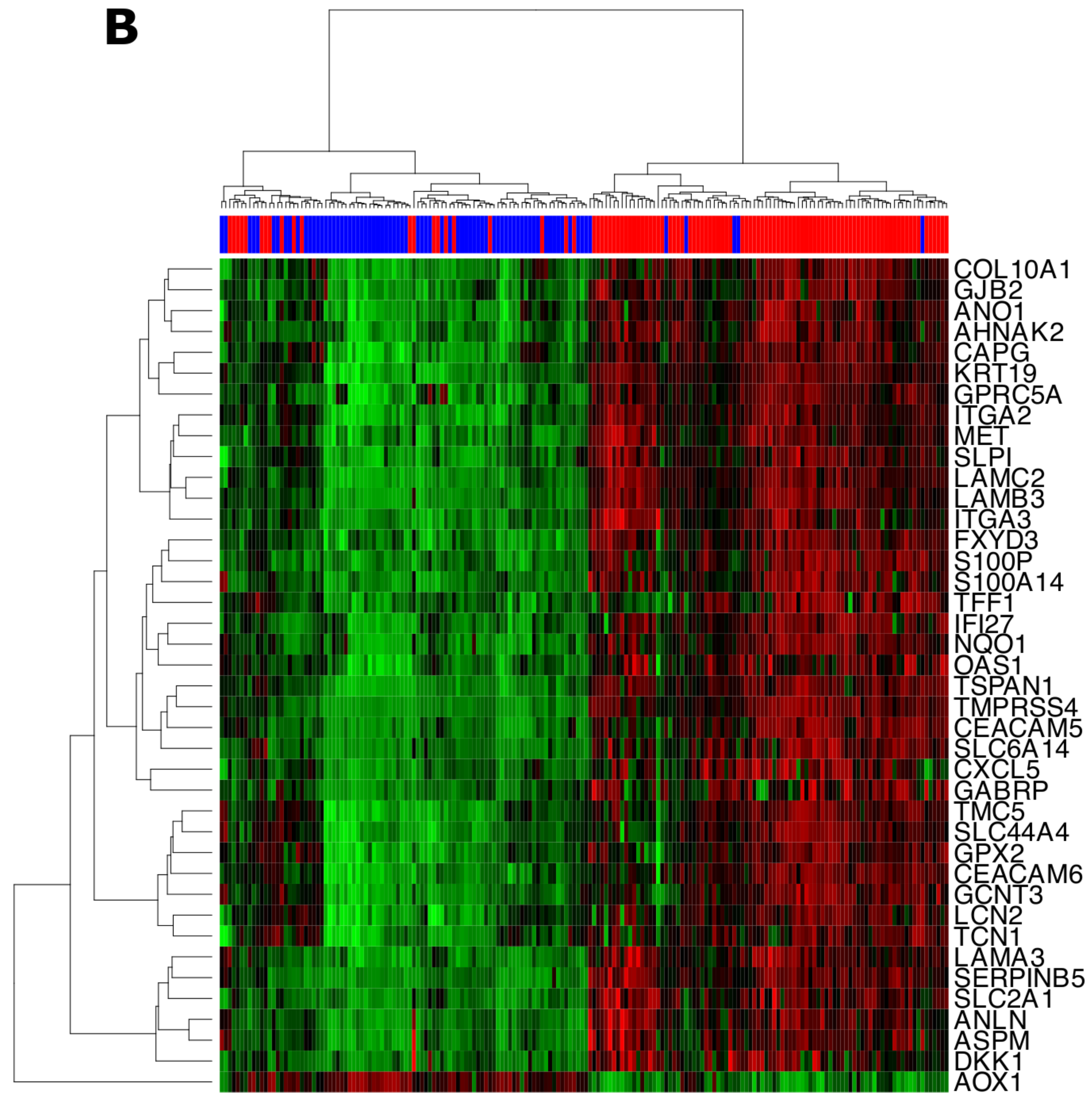**PDAC**

Supplement: Supplementary file 2 — Additional file 2: Figure S1. PCA and hierarchical analysis of the CG expression values from GSE16515. (a) The CG could produce similar results in both PCA and (b) heatmap hierarchical clustering analysis. The CG can classify the data into two groups of normal and tumoral samples. [file 12885_2020_6533_MOESM2_ESM.pdf]

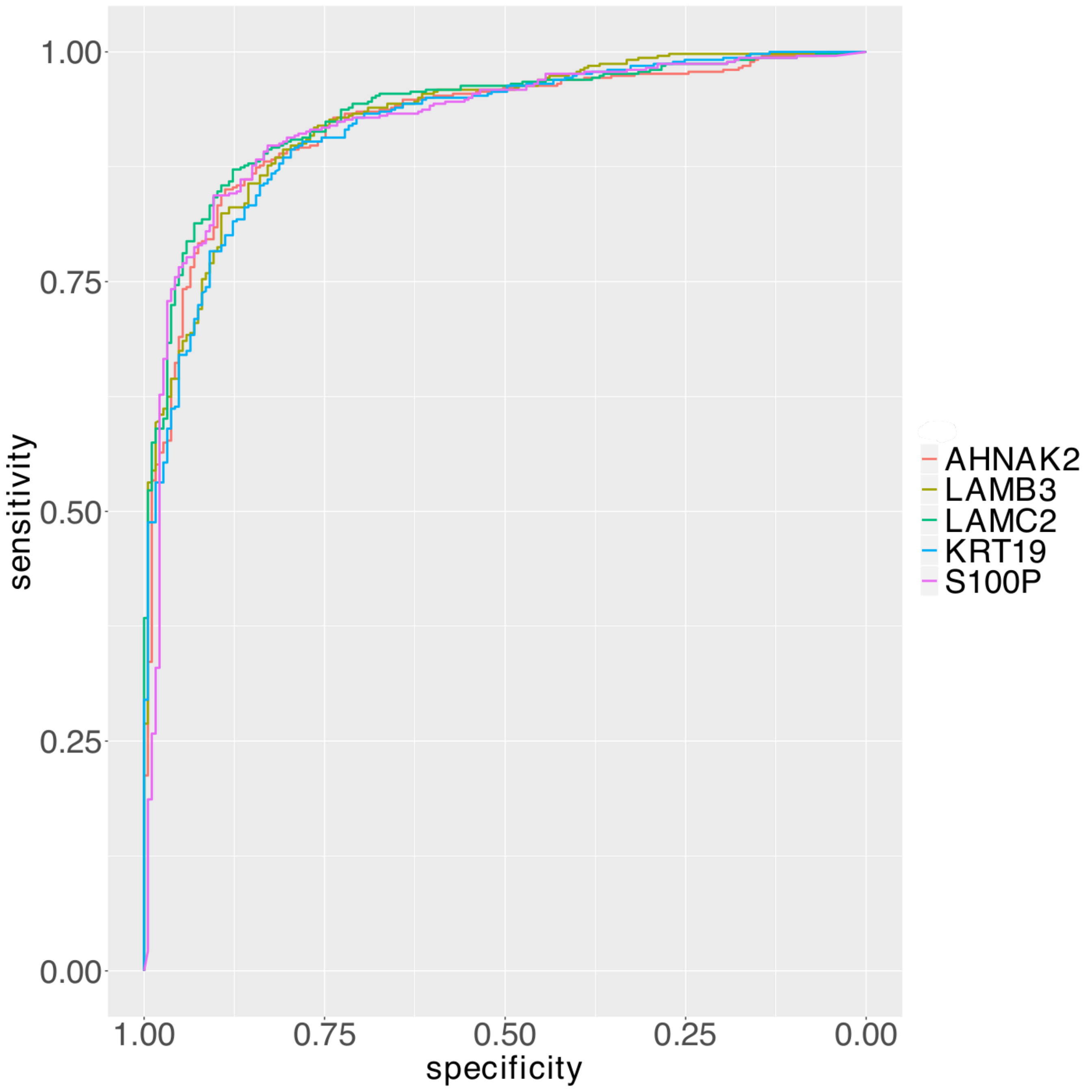

Supplement: Supplementary file 4 — Additional file 4: Figure S2. Receiver operating characteristic (ROC) curve for the five genes selected to train the ANN. The AUC was used to select the genes to train the ANN. The genes selected were AHNAK2 (92.2), KRT19 (92.6), LAMB2 (93.3), LAMC2 (91.8), and S100P (92.3). The AUC for each gene is presented in the parentheses. [file 12885_2020_6533_MOESM4_ESM.pdf]
